# Recent Advances on Thermochemical Recycling of End-of-Life Tires and Their Coblending with Waste Plastic Fractions

**DOI:** 10.1021/acsomega.5c01592

**Published:** 2025-06-18

**Authors:** Ahmad Yaghi, Labeeb Ali, Mohammednoor Altarawneh

**Affiliations:** Department of Chemical and Petroleum Engineering, 11239 United Arab Emirates University, Sheikh Khalifa Bin Zayed Street, Al-Ain 15551, United Arab Emirates

## Abstract

As the number of vehicles on the road continues to rise, so does the challenge of dealing with millions of end-of-life tires (ELT) discarded yearly. These nonbiodegradable materials pose serious environmental and economic concerns, making it crucial to find sustainable ways to recycle them into potential usable products. One of the most promising solutions is pyrolysis, a thermochemical process that breaks down ELT into valuable products. This timely review explores the latest advancements in the pyrolysis of ELT, where different reactor designs were explored, such as fixed bed, fluidized bed, and vacuum reactors, along with key process conditions that influence the quality of products, most notably the tire pyrolysis oil (TPO). The review also examines the copyrolysis of ELT with waste plastics, such as polyethylene, polypropylene, and polystyrene. The primary aim is to enhance the quality of TPO through a series of H-derived reactions. Catalysts also play a crucial role in this process via improving selectivity toward alkylated benzenes, and through reducing the sulfur content for cleaner fuel production. Despite its high potential, pyrolysis of ELT entails considerable drawbacks, most notably the production of polycyclic aromatic hydrocarbons (PAHs) and sulfuric products, which requires advanced control technologies and regulatory frameworks to ensure environmental compliance. By bringing together the latest insights and addressing key obstacles, this perspective highlights the role of pyrolysis-based operations in creating a more sustainable, circular approach to ELT management.

## Graphical abstract

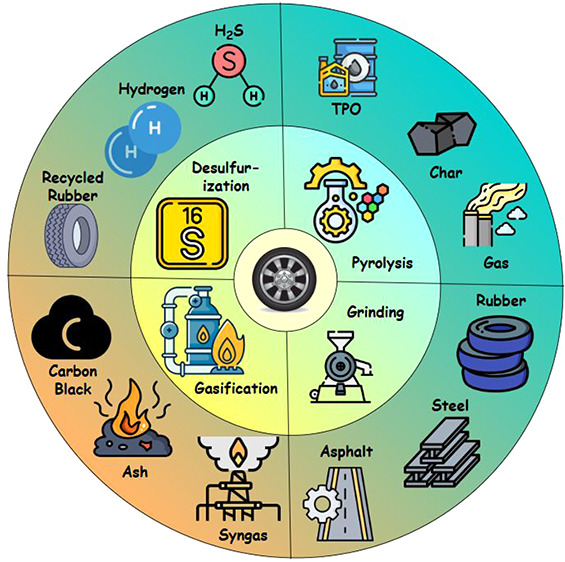

## 1 Introduction

The global production of tires has increased dramatically, reaching an approximate value of 2.5 billion tires produced annually.^1^ Therefore, this corresponds to the disposal of over 1 billion end-of-life tires (ELT) each year, which accounts for approximately 2% of the global waste.^2^ ELT are difficult to handle and store since they occupy a large space and are inherently nonbiodegradable.^3^ The chemical cross-links across the complex polymer chains in tires make the recycling process challenging. Synthetic rubber and natural rubber (NR) in stored tires engender them to be prone to fire hazards.^4^ Accidental fires of tires release a significant load of toxicants into the environment, contaminating environmental matrices.^5^ In landfills, tires are often incinerated under incomplete combustion conditions, a practice that forms notorious pollutants, most notably polycyclic aromatic hydrocarbons (PAHs).^2^ The latter largely resists atmospheric oxidation and thus undergoes long-range atmospheric transport.^5^ It is estimated that 46% of the discarded waste is fully incinerated. Therefore, effective recycling of ELT and their repurposing requires a sustainable strategy that reduces environmental impacts and potentially extracts valuable organic content and black carbon from discarded tires.^6^

Thermochemical recycling of tires via pyrolysis and catalytic pyrolytic upgrading has emerged as a mainstream strategy for their disposal. Pyrolysis constitutes the self-decomposition of a material at an elevated temperature in the absence of oxygen under thermal stress.^7^ The decomposition process typically takes place via different stages, an aspect that reflects the chemical makeup of the material, in which individual components decompose at different temperatures. Several studies in the literature report operational conditions and products that arise from the pyrolysis of tires and their copyrolysis with plastic fractions.^8^ The latter simulates scenarios involving heterogeneous waste mixtures that entail the presence of complex waste mixtures.^9^ During pyrolytic degradation of tires, several components interact (rubber, silica, S-containing species, and carbon black),^10^ rendering the underlying mechanism rather complicated.^11^

The copyrolysis of ELT with other solid waste (i.e., plastic and biomass) has been investigated with two main broad aims: to improve the quality of the produced pyrolytic oil and to mimic representative real waste mixtures where separation of various waste stocks is an expensive exercise.^12^ The main materials that can be copyrolyzed with the ELT are polymers that contain high hydrogen contents, most notably, polyethylene (PE), polystyrene (PS), polypropylene (PP), and other kinds of polymers.^13^ The use of catalysts in the secondary upgrading process enables two types of reactions to take place, hydrogenation and β C–C fission reactions.^14^ The applied catalytic upgrading processes alter the product distribution and improve the selectivity toward the desired products.^15^

Evaluating the most optimum routes for recycling of ELT requires a life cycle assessment of the different tire production stages. Raw material extraction is the main stage, which consumes high energy and produces emissions from synthetic rubber.^16^ Involved operations such as mixing, molding, and finishing require high energy usage and emit volatile organic compounds (VOCs). After manufacturing, the tires will be ready to be installed on vehicles, where, after long usage, they become ELT that require recycling. In addition to thermochemical operations, mechanical-based options have been utilized to treat the ever-increasing number of ELT. For instance, recycling the ELT into crumbs for asphalt applications can reduce the CO_2_ emissions and enhance sustainability. As such, recycling of ELT align with several UN Sustainable Development Goals (SDGs), such as promoting innovative technologies for recycling (SDG #9), reducing pollution and greenhouse gases (GHG) emissions through management (SDGs #11 and #13), and encouraging the recycled material usage (SDG #12).^16^

To this end, this survey aims to present recent advances pertinent to the pyrolysis of ELT and their copyrolysis with common plastic wastes. The review discusses different aspects and parameters that affect the reaction mechanisms and products and presents challenges, limitations, and potential future directions. Important aspects related to copyrolysis of tires with plastic waste are thoroughly discussed, with a prime focus on the mixing ratio, reactor types, operational conditions, and product yields. It is often illustrated that the type of added plastic waste (PE, PS, and PP) and the reactor’s design entail a noticeable influence on the composition of pyrolytic products. Moreover, catalytic upgrading processes are reviewed to comprehend how various active metals/supports lead to the production of distinct reaction profiles and how to design an effective catalytic process with the governing objective being to enhance the quality of the attained oil and to reduce the energy requirements of the pyrolytic operation. Perspectives on real-scale thermochemical operations are discussed. The review concludes by presenting potential future directions and concluding remarks. Sections and scope of the review paper are shown in Figure 1.

**Figure 1:**
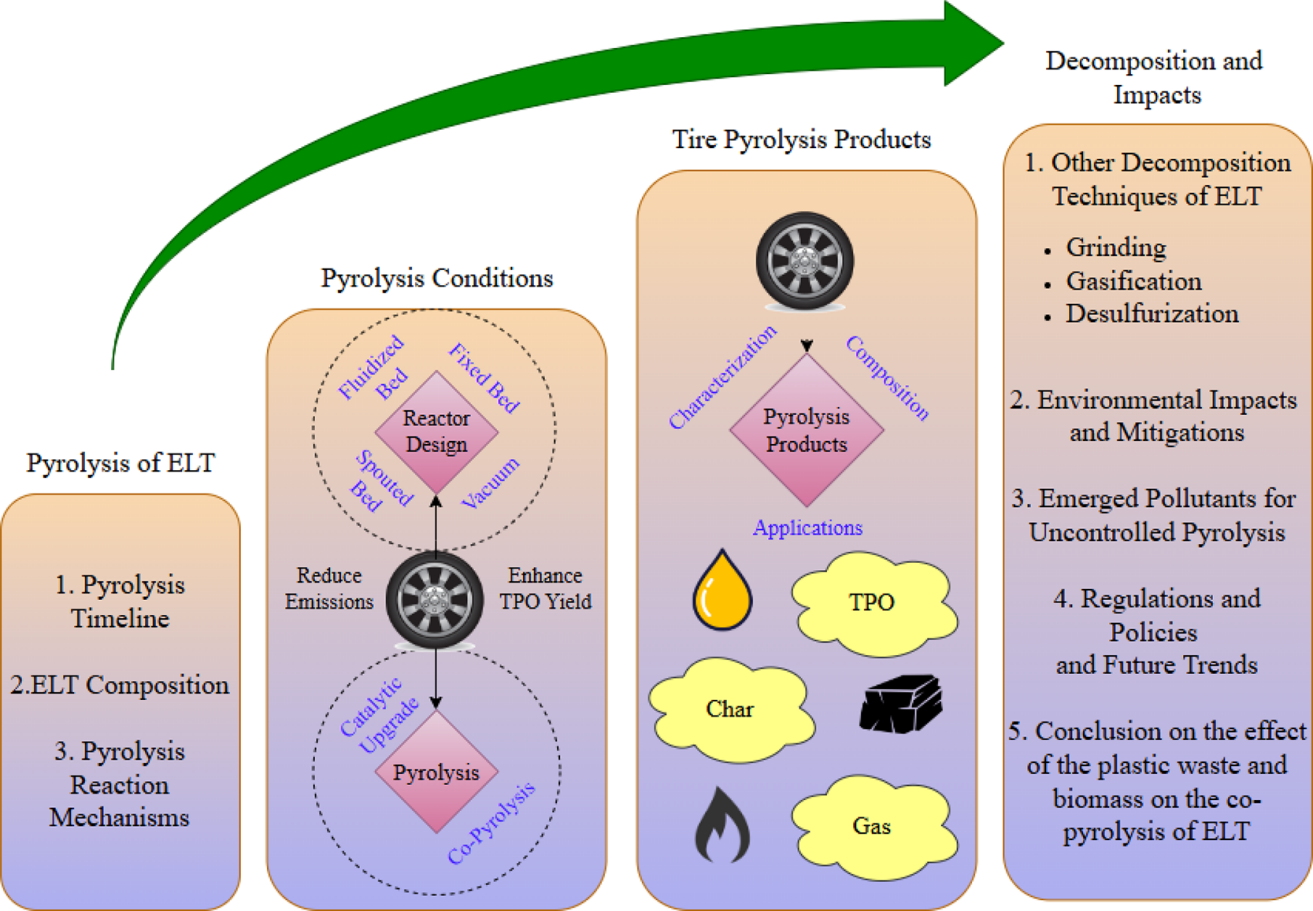
Sections and scope of the review paper.

## 2 ELT Recycling

### 2.1 Timeline of ELT Pyrolysis

Recognizing the large amount of waste produced by ELT and the unprecedented challenge to dispose it, a great deal of applied and fundamental research has focused on pyrolytic degradation of waste tires since the 1980s. Interest in the pyrolysis of discarded tires also originated from the high energy required to produce fine particles from their grindings.^1^ According to Scopus, Figure 2 specifies the number of published articles on the topic of recycling ELT from the early 1990s to present, while Figure 3 collates the most important keywords in these articles. Interestingly, the number of articles on the pyrolysis of waste tires positively correlates with the linear increase in the number of vehicles worldwide (between 2020 and 2024).^17^ As displayed in Figure 3, the pyrolysis of tires is often discussed in the context of “sustainability,” “circular economy,” and “life cycle assessment.” This indicates that pyrolysis of discarded tires entails important socio-economic aspects that tap into several UN SDGs, most notably SDG # 11 (sustainable cities and communities) and SDG # 12 (responsible consumption and production). Other important technical keywords include “gasification,” “desulfurization,” and “catalytic pyrolysis.” While waste management of discarded tires seems a purely technical issue, it is also interwind with important social, health, sustainability, economic, and environmental dimensions.

**Figure 2:**
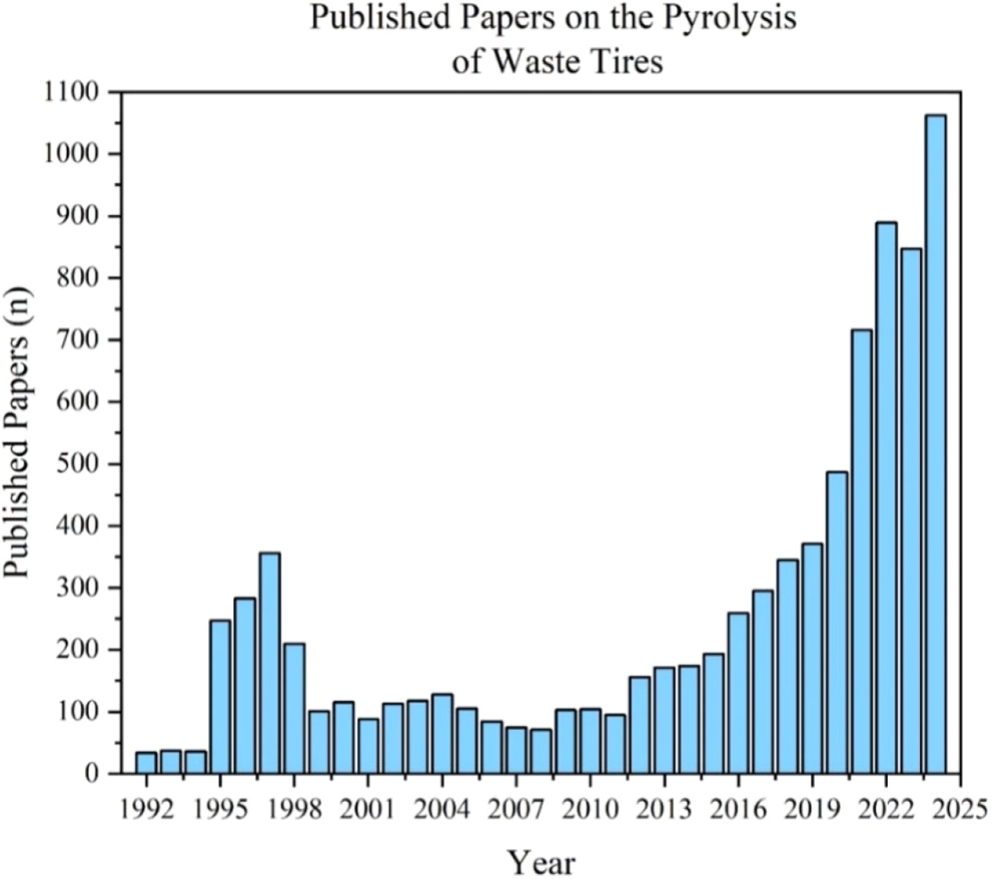
ELT pyrolysis published articles according to Scopus.

**Figure 3:**
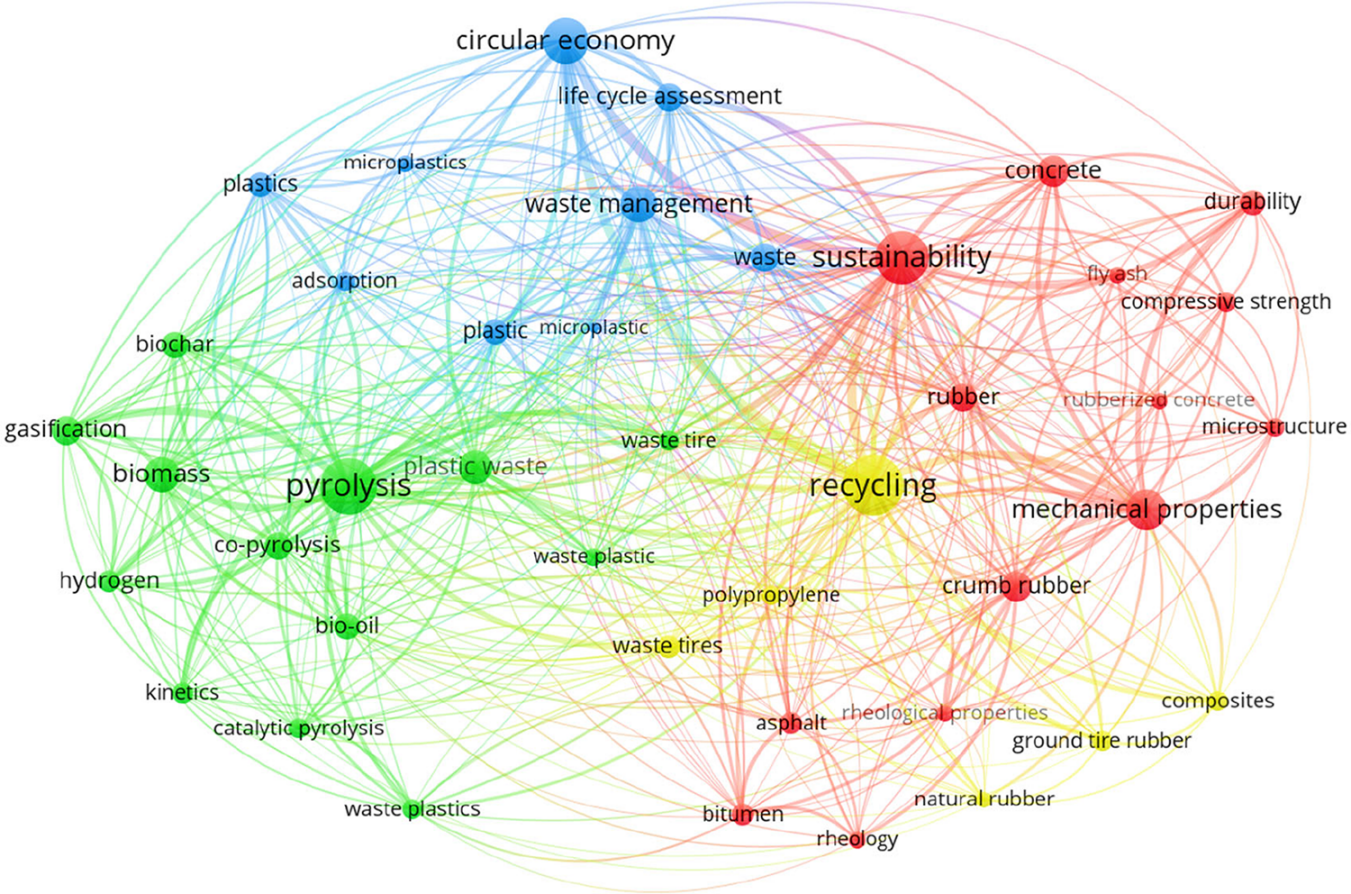
Main keywords and their connections from articles that investigate pyrolysis of ELT between 2019 and 2024, as extracted from Scopus.

### 2.2 ELT Compositions

Tires are composed of different categories of rubbers and other materials that are added to enhance the rubber’s properties and strength. Figure 4 displays the typical compositions of tires and ELT.^18^ First of all, tires are made mostly from various types of natural and synthetic rubbers, which contain a variety of polymers and monomers, most notably, butadiene, styrene, propylene, and isobutene.^19^ Continuous friction, exposure to heat, sunlight, and atmospheric oxygen, and moisture content gradually remove the outer rubber layer during the lifetime of the tire. Therefore, ELT lose ∼2 to 3% of its initial rubber content. In addition to the rubber content, carbon black constitutes the main component of discarded tires (i.e., 30% by mass). Black carbon is added to enhance the strength and durability of the tires. Additives like silica and clay are used as a replacement for carbon black, while metals like steel (13–16%) and textiles (5–6%) are used for reinforcing the tire and increasing its strength. A small percentage of sulfur (1–2%) is used to harden the rubber and prevent any potential deformation.^20^ The same components that afford tires with profound mechanical and structural stabilities also render it very challenging and energy intensive to grind or chemically dispose of used tires.

**Figure 4:**
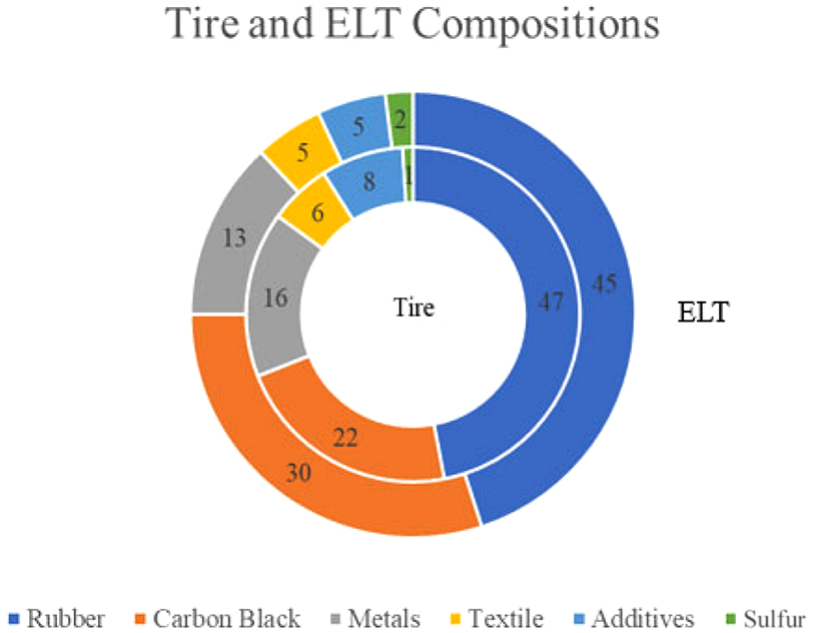
Tire and ELT compositions.^17^ The inner and outer circles denote the composition of tires and ELT, respectively.

### 2.3 Pyrolysis Reaction of ELT

Under pyrolytic conditions, the chemical bonds that connect the building blocks in discarded tires undergo fission reactions to initially release monomeric building blocks.^18^ Secondary unimolecular and bimolecular reactions accelerate the decomposition process. The absence of oxidizing agents in the reaction medium promotes the formation of single-aromatic compounds and shuts down pathways that form CO_*x*_ gases.^21^ Fragmentation of the three rubber building blocks in tires, namely, butadiene rubber (BR), NR (polyisoprene), and styrene–butadiene rubber (SBR), initiates the formation of fuel-like products, most notably, 1,3-butadiene, styrene, xylene, and benzene.^21^ Figure 5 depicts the postulated reaction pathways that govern the thermal degradation of reaction (SBR) in which alkylated benzene emerges as the main product at high temperatures. Three types of reactions generally ensue: dehydrogenation, intramolecular cyclization, and dealkylation. Dealkylation reactions starting from d-limonene form benzene-xylene-toluene (BXT) compounds. Likewise, toluene is predicted to emerge from the hydrogenation of styrene’s double bond. Toluene serves as the building block for styrene through the addition of a methyl group at the meta site. Further degradation reactions of BR produce 1,3-butadiene. Hydrogenation and decomposition reactions of the latter produce simple hydrocarbons, alkanes, or alkenes. 1,3-butadiene also acts as a precursor for cyclic compounds such as 1,3-cycloheptadiene or 4-vinyl-1-cyclohexene.^7^ Degradation of NR produced D-limonene after going through intramolecular cyclization where the C–C bond ruptures to produce alkatriene that goes into cyclization to produce various cycloalkenes. These cycloalkenes go through dehydrogenation to produce the BTX compounds. Limonene can also produce *p*-cymene when hydrogenated, which produces the simple BTX compounds after dealkylation reactions.

**Figure 5:**
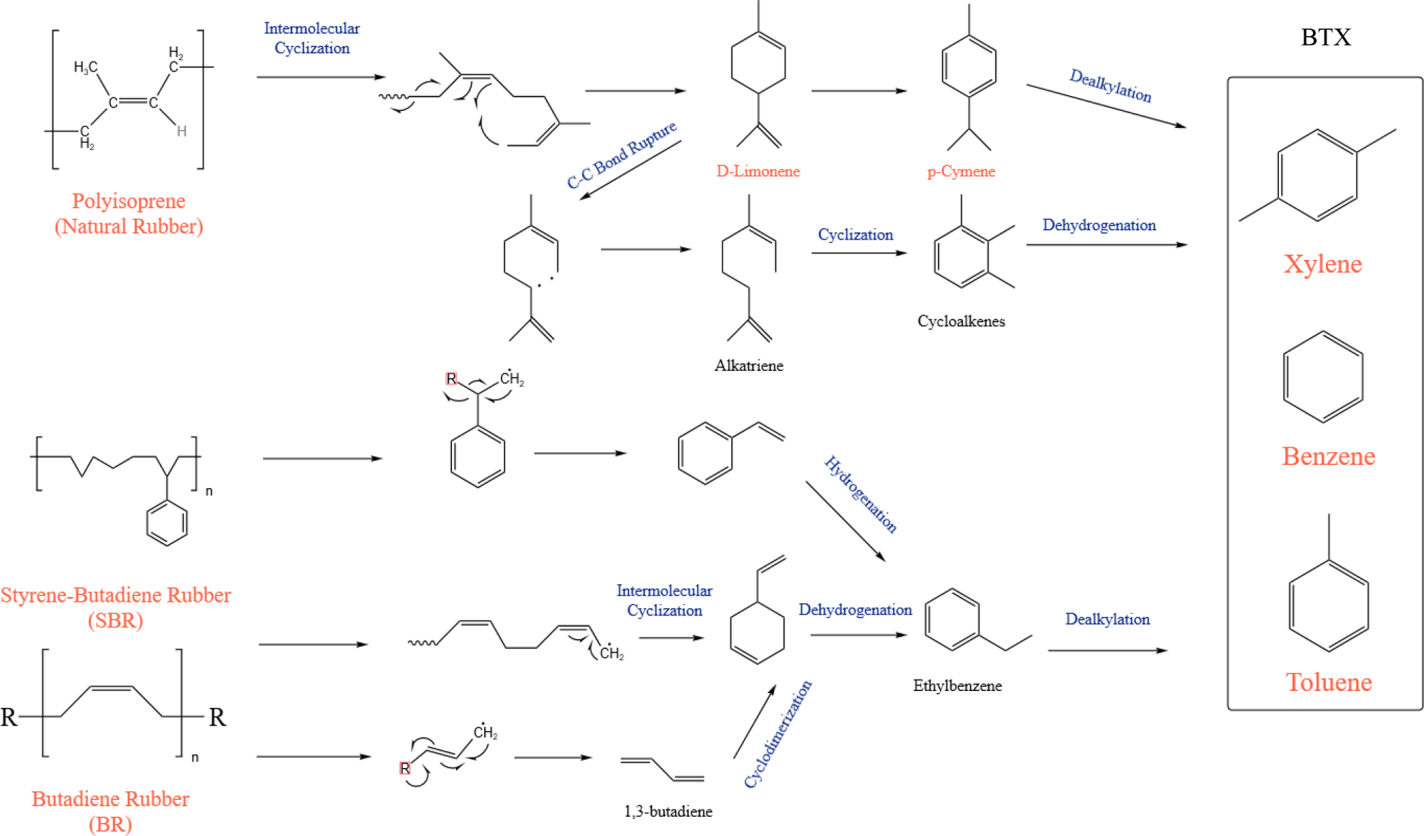
Chemical reactions that govern the degradation of ELT.

## 3 ELT Pyrolysis Conditions

### 3.1 Pyrolysis Reactor Design

Detailed specifications of the main pyrolysis reactor commonly used in ELT thermochemical conversion are provided in the literature;^22^ herein, we refer to the salient operational conditions and yields of TPO.

#### 3.1.1 Fixed Bed (Batch)

Fixed bed reactors have been utilized for the pyrolysis of ELT by numerous scholars due to their ease of handling and operation. Figure 6 shows the maximum yield of the pyrolysis products from the reaction that was performed by using fixed bed reactors at different operating temperatures. For instance, Banar et al.^23^ investigated the pyrolysis of ELT in a fixed bed reactor, where it was suggested that using a heating rate of 5–35 °C/min for the pyrolysis produces the maximum products at 400 °C. These products correspond to tire pyrolysis oil (TPO), char, and gas, where TPO had the maximum yield compared to the other products. Along the same line of enquiry, Islam et al.^24^ considered the same reaction but with different heating rates and conditions. Islam et al.^25^ recycled ELT with a fixed bed reactor provided with internal fire tubes, where the highest yield of TPO was produced at 450 °C. On the other hand, Ilkilic et al.^26^ and Laresgoiti et al.^27^ produced the maximum yields of TPO at higher temperatures, reaching 500 and 700 °C, respectively.^28^ This noticeable difference in temperature is attributed to varying reaction conditions, in terms of applied heating rates, compositions of the tires, and pretreatment parameters. However, the relative loads of the product distribution among the three phases follow similar trends even as the temperature increases.

**Figure 6:**
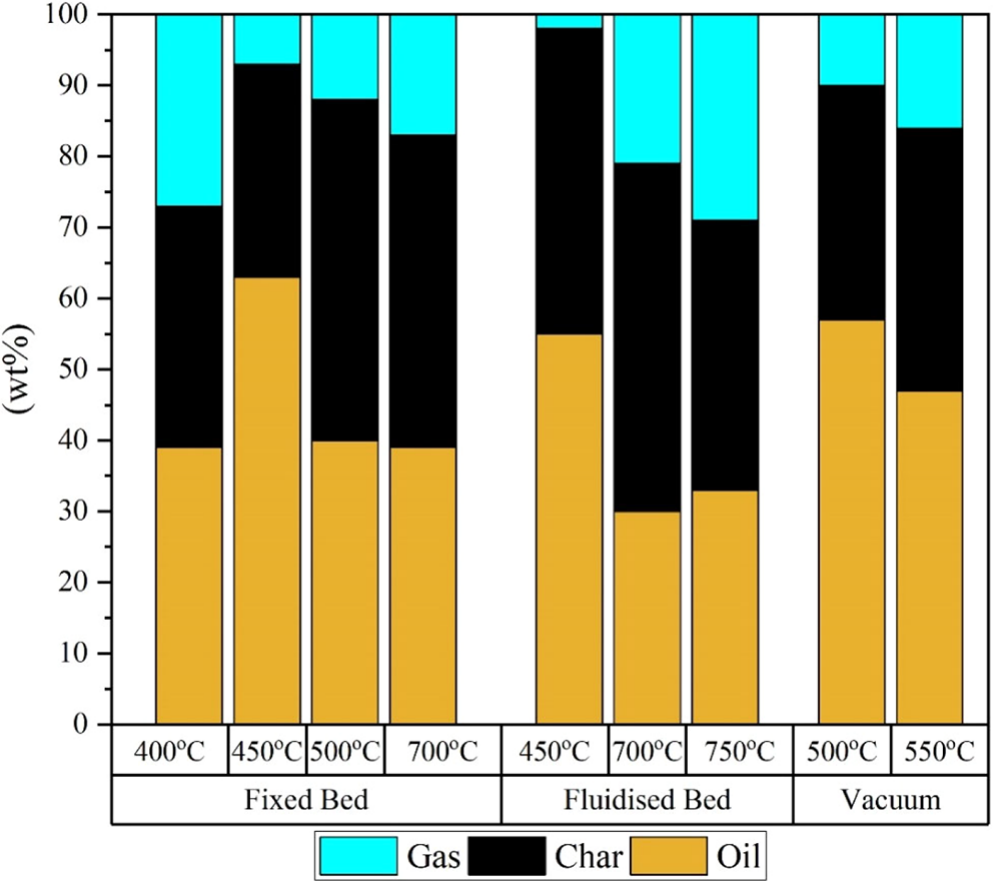
Temperature-dependent phase distribution of products from the pyrolysis of ELT using fixed bed,^23,25,28^ fluidized bed data,^29–31^ and vacuum reactors.^33,34^

#### 3.1.2 Fluidized Bed

Fluidized bed constitutes the commonly deployed reactor in the pyrolysis of ELT. Three experiments were graphed in Figure 6. Williams and Brindle^29^ studied the pyrolysis of shredded ELT at a flow rate of 220 g/h, where the TPO yield peaked at 450 °C; moreover, their products entail low gas quantity. The same aspect also prevails when the pyrolysis reaction was carried out at the fixed bed reactor. In addition, Kaminsky et al.^30^ focused on high-temperature pyrolysis with various reaction conditions and loads. The primary experiment used a whole tire with a temperature of 700 °C, where the products were mostly char with negligible formation of gas and liquid products. Products from shredded tires share similar features. As Figure 6 depicts, products from the pyrolytic decomposition of ELT in a fixed bed reactor share similar phase distribution to their analogous values attained in fluidized bed.^31^

#### 3.1.3 Spouted Bed Reactor

The spouted bed reactor is an emerging reactor type specifically used for the pyrolysis of ELT, which gained increasing attention due to its design and efficiency, rapid heat transfer, and improved handling of large particles such as shredded tires.^32^ Moreover, this type of reactor avoids bed agglomeration issues; thus, it performs very well in processing rubbery materials. Studies reported that the pyrolysis of ELT using the spouted bed reactor offers high TPO yields and efficient thermal distribution, which will reduce energy consumption and yield high-quality products.^32^ Additionally, this reactor operates for continuous operations, making it suitable for mimicking industrial-scale applications. López et al.^32^ utilized the spouted bed reactor between 425 and 600 °C to attain a high TPO yield of 44.5–55.0 wt %. The improved performance of the spouted bed reactor arises from cyclic movement that enhances contact between phases.^32^

#### 3.1.4 Vacuum

A vacuum reactor is an additional reactor used for pyrolysis purposes of ELT, but it is not commonly used in this application. The significant advantage of the vacuum reactor is that it decreases the pyrolysis temperature while improving the quality of TPO and its yield. Moreover, it operates under a vacuum pressure that limits the emissions and odors that are produced from the reaction. Pakdel et al.^33^ constructed a pilot-scale plant based on the vacuum reactor to investigate the pyrolysis of ELT at 500 °C. As Figure 6 shows, a relatively higher load of TPO was attained when compared with the fluidized bed reactor. A study by Zhang et al.^34^ using a batch vacuum reactor at 550 °C attained a similar product distribution to those depicted in Figure 6.

#### 3.1.5 Comparative Performance of the Pyrolysis Reactors

Table 1 compares the performance of the various types of pyrolysis reactors in terms of the TPO yield. Batch reactors entail the lowest TPO yield at ∼20%. On the other hand, the maximum TPO content was obtained using a conical bed reactor. This improvement is primarily attributed to the reactor’s geometry, which enhances heat distribution and vapor residence time, promoting more efficient pyrolysis reactions and higher oil yields.^35^ TPO yields from fixed bed reactors were comparatively high compared to that attained in conical bed reactors. The same goes for the yield of the gaseous products. Looking at the maximum temperatures of these reactions, the data indicates that higher yields of oils are produced at a temperature range of 400–500 °C,^18^ regardless of other reaction conditions such as the heating rate, the residence time, and the type of feedstock.^35^

**Table 1:** Phase Distributions of Products from the Pyrolysis of ELT Using Different Reactors

		maximum product yield			
reactor	temperature	oil (wt %)	char (wt %)	gas (wt %)	ref
batch	388 °C	19.4%	2.6%	3.2%	^36^
batch	505 °C	17.5%	6.9%	29.7%	^36^
batch	505 °C	13.1%	22.6%	7.8%	^36^
conical bed	500 °C	66.6%	22.2%	9.9%	^37^
conical bed	500 °C	61.8%	27.1%	10.7%	^37^
fixed bed	400 °C	38.8%	34.0%	27.2%	^23^
fixed bed	450 °C	55.0%	36.0%	9.0%	^25^
fixed bed	500 °C	40.26%	47.88%	11.89%	^28^
fixed bed	700 °C	38.5%	43.7%	17.8%	^27^
fluidized bed	450 °C	55.0%	42.5%	2.5%	^29^
fluidized bed	700 °C	26.8%	35.8%	19.0%	^31^
fluidized bed	750 °C	31.9%	38.0%	28.5%	^30^
Parr	400 °C	47.9%	34.1%	18.0%	^38^
vacuum	500 °C	56.5%	33.4%	10.1%	^33^
vacuum	550 °C	45.0%	36.0%	6.0%	^39^
vacuum	550 °C	47.1%	36.9%	16.0%	^34^
spouted bed	550 °C	55.0%	38.2%	6.8%	^32^

### 3.2 Copyrolysis of ELT with Plastic Waste

In real scenarios encountered in waste management facilities, ELT are not entirely pure when recycled as these materials are often mixed with other waste. Plastic waste is among the most prolific solid waste worldwide.^40^ As such, it is important to investigate copyrolytic interactions of discarded tires with plastic wastes such as PE, PP, and polyvinyl chloride (PVC).^13^ Unlike components of waste tires, a fraction of plastic waste entails high H/C fractions;^41^ as such, copyrolysis of these two polymeric waste streams is anticipated to alter the composition of products in reference to the pyrolysis of discarded tires. In addition to plastics, pyrolytic mixtures of discarded tires with biomass^42^ and coal^38^ were also investigated. A consensus of opinions in the literature indicates that copyrolysis of plastic wastes with ELT enhances the chemical makeup of TPO^42^ via improving its content of fuel-like compounds such as benzene, toluene, and other high-quality TPO.^13^ For instance, it has been recently demonstrated that interactive pyrolysis of ELT with PS effectively eliminates the sulfur content from TPO.^43^ The working hypothesis is that H atoms generated from the degradation of the polymeric constituents in plastic interact with S moieties, converting them to evolved gaseous H_2_S.

#### 3.2.1 Copyrolysis Mass Loss

To illustrate the effect of the addition of plastic waste on the pyrolysis of ELF, the reduction in the amount of leftover residue (i.e., char) often serves as an indicator. As Figure 7 demonstrates, copyrolysis of ELF with PS, PVC, and PE systematically reduces the char amount, which accumulates at the end of the degradation process.^42^ For instance, the amount of char from the pyrolysis of neat ELF stands at ∼42% of the initial mass. Addition of PE reduces the unburnt mass to ∼15% at 423 °C. Similar effects were observed upon the addition of PVC and PS to ELF. Combining PVC had the same effect on tires, where the maximum loss decreased at higher temperatures. On the other hand, PE had the maximum loss compared to other combinations, but even higher temperatures were required to produce this outcome.

**Figure 7:**
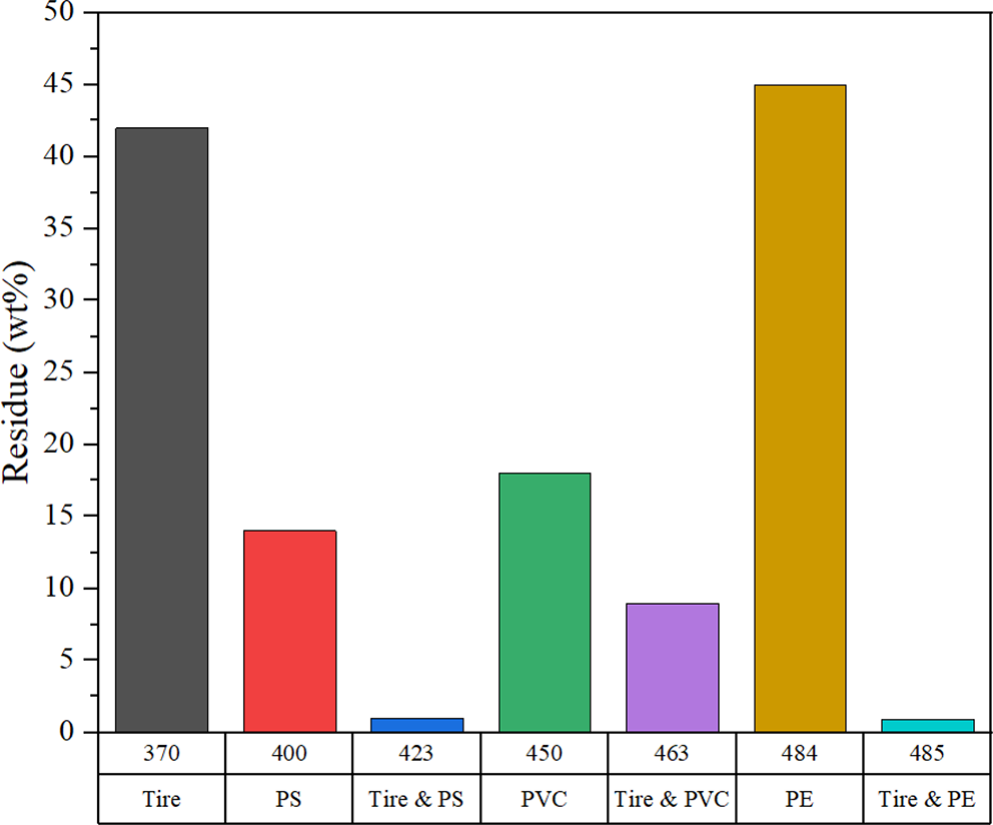
Pyrolysis of ELT with different plastic waste. Values in the *x*-axis denote temperatures in K. Data were reproduced from ref 13.

#### 3.2.2 Enhancement in the Yield of TPO upon Copyrolysis of ELT with Plastics and Biomass

Table 2 summarizes selected studies that report the yield of TPO from the copyrolysis of ELT with various fractions of plastics (mainly PS, PP, and PVC) and biomass. The yield of TPO consistently increased upon the addition of plastic and biomass components. For instance, copyrolysis of ELT with PP increased the TPO from 30% to ∼85%. The increase in the yield of TPO was enhanced by more than 75% when PS, PE, and PVC were added individually to ELT. The addition of PET, in particular, increased the production of gaseous products dramatically compared to the other plastics at the same temperature.^42^ The maximum char production was found using PVC at 520 °C. This proves that these different plastics can alter the reaction mechanism to yield different products of various qualities and compositions. The copyrolysis of tires with agricultural waste or biomass was also investigated. Table 2 outlines several of findings from selected copyrolysis studies with a focus on the phase distribution. Palm shell and sawdust produced a high yield of char as reported by Kumar et al.^44^ and Li et al.^13^ On the other hand, pine sawdust assumes a different copyrolytic reaction, yielding a high quality of oil with a weight of 66.6%, which demonstrates the difference between sawdust and pine sawdust and their effect on the copyrolysis of tires. On the other hand, copyrolysis with microalgae attained the maximum yield of gaseous products as reported by Kordoghli et al.^36^ Last, using mixed coal with tires produced other components, such as steel and other chemical compounds, without producing char and gas.

**Table 2:** Summary of Different Materials Used for Tire Copyrolysis, with the Product Yield

		maximum product yield			
material (copyrolysis)	temperature	oil (wt %)	char (wt %)	gas (wt %)	ref
PS	450 °C	69.67%	20.17%	7.61%	^42^
PP	505 °C	73.5%	18.3%	12.8%	^42^
PE	490 °C	70.50%	16.50%	13.94%	^13^
PVC	520 °C	66.6%	22.2%	9.9%	^42^
PET	510 °C	56.33%	21.67%	24.58%	^13^
palm shell	500 °C	46.2%	36.4%	17.4%	^44^
pine sawdust	500 °C	66.6%	22.2%	9.9%	^37^
mixed coal	400 °C	36%			^45^
microalgae	550 °C	46.8%	31.5%	21.6%	^46^
sawdust	450 °C	32.0%	50.1%	17.6%	^47^

To further understand the effect of plastics on the pyrolysis of tires, Figure 8 offers an insight. The pyrolysis of tires alone produces a considerable number of components in the TPO fraction with a reduced quantity of gases and char, while including PP in the tires reduces gases and increases the yield of char. The addition of PS had the same effect, with even higher production of char, while combining of ELT with PVC led to different results by increasing the production of gases while producing less amount of char.^42^ The maximum production of gases was observed with the addition of PET. Therefore, this shows how these plastics can alter the products of the pyrolysis process of tires. Mechanistically, fragments from plastic fractions promote degradation of ELT components, thus enhancing the TPO and gas yields.^13^

**Figure 8:**
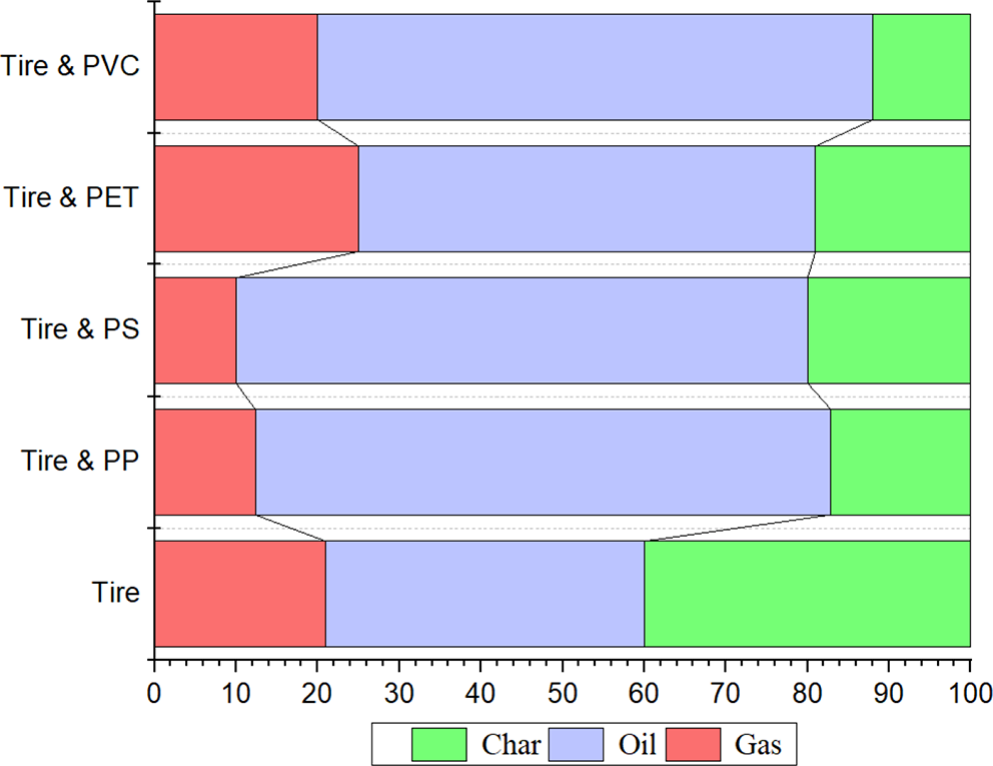
Products of the copyrolysis of ELT using different plastic waste. Data related to addition of PS, PP, and PVC were sourced from ref 42, while those of PE and PET were taken from ref 13.

### 3.3 Catalytic Pyrolysis of ELT

The effect of catalysts on the pyrolysis of ELT has been investigated to some extent.^48^ Catalysts with acidic nature such as zeolite were found to improve the quality of TPO. As Figure 9 shows,^49^ acidic catalysts promote the formation of monoaromatic hydrocarbons during pyrolysis of ELT and increase the TPO yield, mainly via surface-mediating Diles-Alder type reactions starting from unsaturated C_4_ cuts.^49^ Other additives such as calcium hydroxide [Ca (OH)_2_] increase the production of char and decrease the gas outcome of the reaction, which is the same when using zeolite (ZSM-5) as a coadditive.^50^ As in other catalytic upgrading operations, different catalysts mediate distinct reaction pathways.^5^

**Figure 9:**
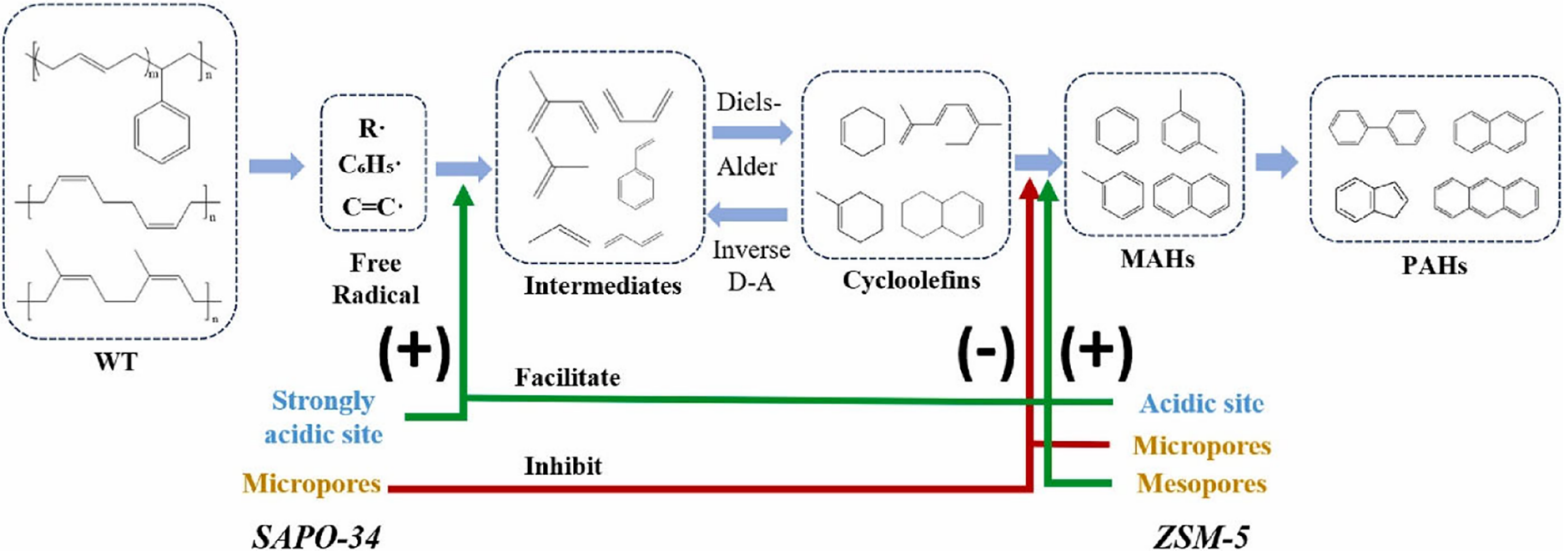
Effect of acidic catalysts on the pyrolysis products of ELT. Adapted with permission from ref 49 copyright [2024] ELSEVIER.

Table 3 summarizes the phase’s fractioning from catalytic upgrading of ELT’s pyrolysis over with various types of catalysts.^36^ Pertinent results are also portrayed in Figure 10.^26,51,52^ It is generally found that the addition of these catalysts to the pyrolysis of tires promotes degradation of the char and TPO content into gaseous products.^5^ Nonetheless, it is also viewed that in addition to the type of catalysts, the relative yields of phases (char, TPO, and gas) are sensitive to the pyrolysis conditions, most notably the type of the reactor and the load of the materials (additives and ELT).^53^ The catalysts' reactivity and their origin are comprehended by various characterization techniques that underpin the nature of the active sites, their dispersion, and geometrical and electronic attributes.^36^ Figure 11 shows the sample characterization results of catalysts commonly used in the upgrading of TPO.^54–56^

**Figure 10:**
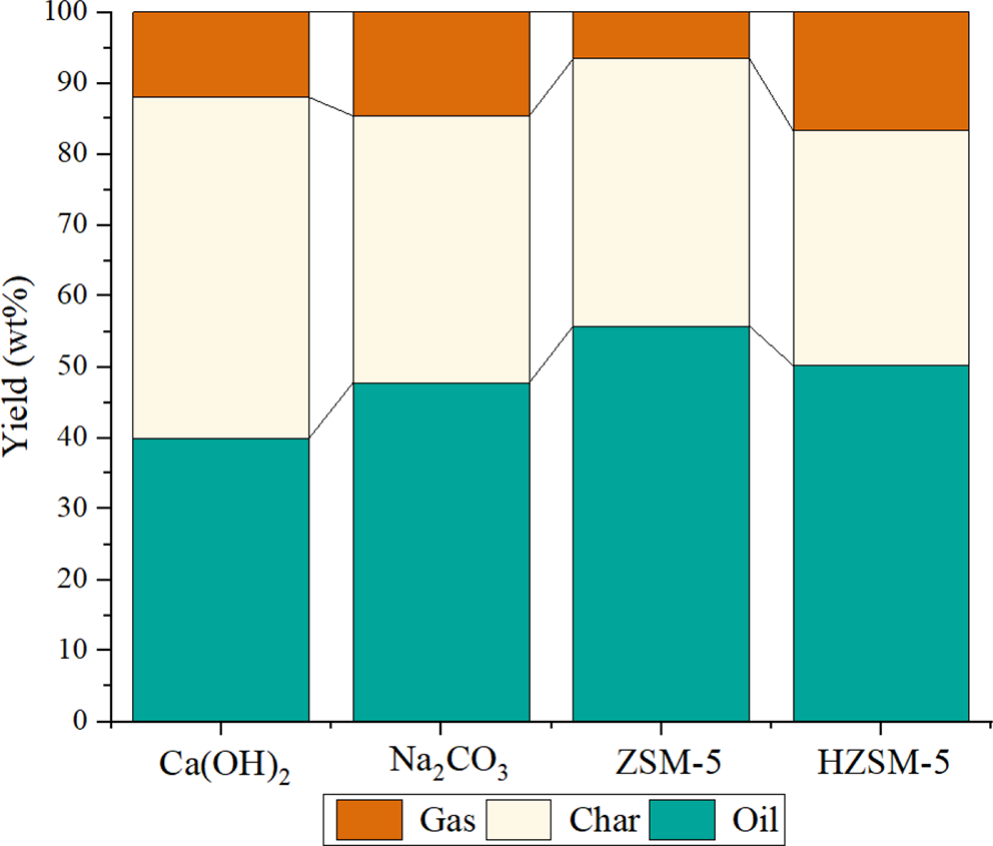
Phase distributions of products from the pyrolysis of ELT. Data related to Ca (OH)_2_, ZSM-5, and Na_2_CO_3_ were sourced from refs 26, 51, and 52 , respectively.

**Table 3:** Summary of Catalytic Pyrolysis of Tires Using Different Catalysts, with their Yield

		maximum product yield			
catalyst	temperature	oil (wt %)	char (wt %)	gas (wt %)	ref
ZSM-5	500 °C	55.60%	37.6%	6.5%	^53^
ZSM-5	500 °C	30.0%	44.8%	25.5%	^36^
ZSM-5	600 °C	35.83%	47.1%	12.1%	^51^
Al_2_O_3_	500 °C	32.5%	37.4%	30.0%	^36^
Al_2_O_3_	450 °C	34.5%	36.6%	36.6%	^57^
Na_2_CO_3_	600 °C	49.2%			^52^
Na_2_CO_3_	500 °C	47.8%	37.6%	6.50%	^52^
MgO	400 °C	39.8%	35.8%	24.4%	^58^
CaCO_3_	400 °C	32.23%	35.23%	32.53%	^59^
Ca (OH)_2_	500 °C	40.0%	48.0%	12.0%	^26^
Ru/MCM-41	600 °C	24.8%	44.50%	32.0%	^60^
Cu/HBETA	500 °C	39.0%	40.50%	39.0%	^61^

**Figure 11:**
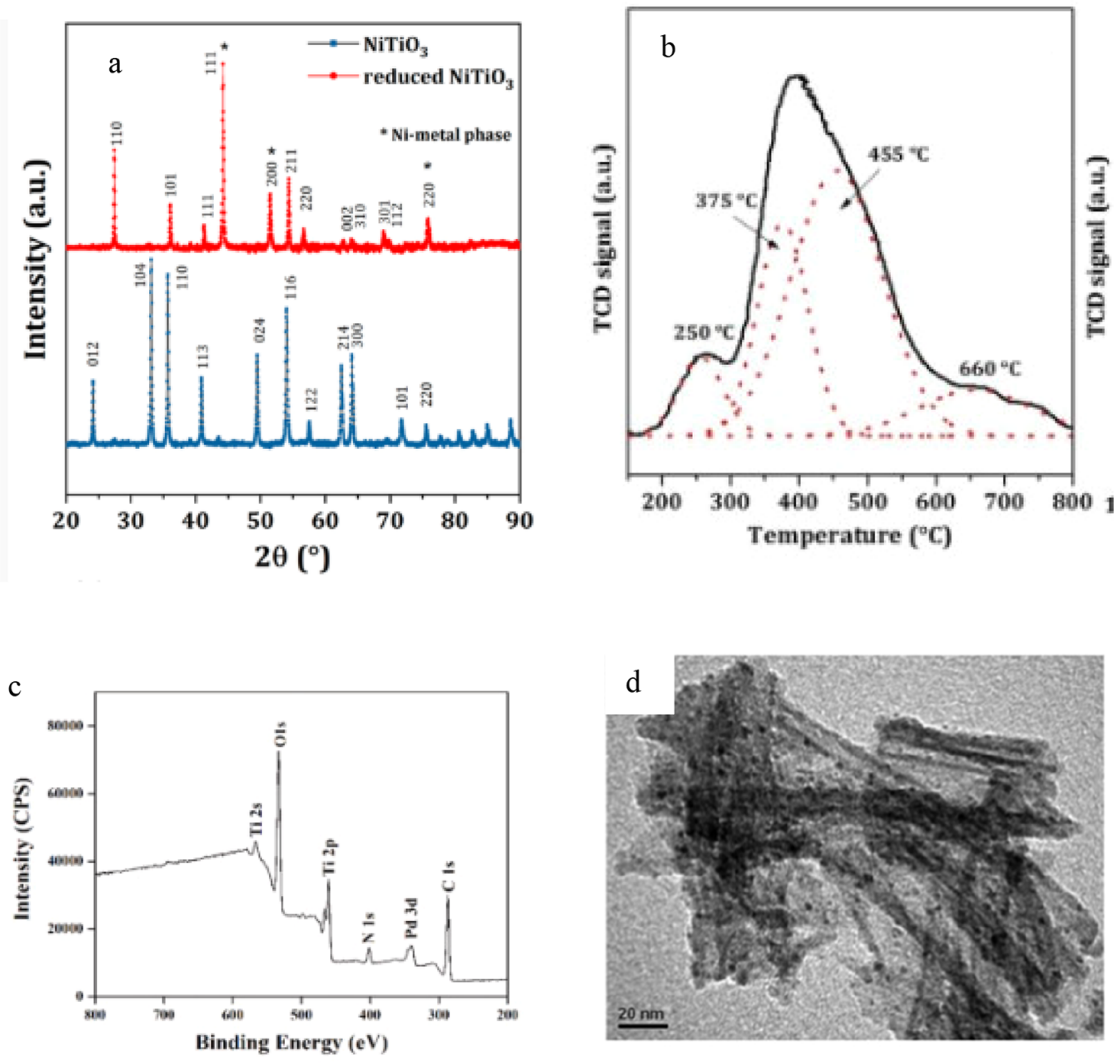
(a) X-ray diffraction patterns for ilmenites-based catalysts of Ni adapted with permission from ref 54 copyright [2022] MDPI. (b) Temperature-programmed reduction profiles of Ni adapted with permission from ref 55 copyright [2021] ELSEVIER. (c) X-ray photoelectron spectra of Pd 3d for the as-synthesized Pd/NT-T. (d) TEM micrographs of Pd/NT-Ti. Both are adapted with permission from ref 56 copyright [2022] ELSEVIER.

## 4 Tire Pyrolysis Products

Besides reducing the volume of discarded tires, the main aim of the pyrolysis of ELT is to produce TPO that is rich with fuel-like compounds, most notably BXT compounds.^18^ Previous sections discussed the yields of the TPO and the phase distribution into char and gas in three copyrolytic systems, neat ELT, plastic-blended ELT, and catalytic upgrading of ELT’s TPO. Herein, the chemical composition of the TPO and its further treatment approaches are discussed.

### 4.1 TPO Product Analysis

#### 4.1.1 TPO Compounds

Thermochemical conversion of the rubber materials in ELT produces TPO in addition to the gas fraction.^62^ The char mainly originates from the back carbon. The chemical composition of TPO features a very complex mixture with a wide array of aliphatic and aromatic compounds.^63^ The aliphatic fraction contains alkanes or alkene-like decane, hexene, or octene.^64^ Furthermore, the primary products formed during the pyrolysis of ELT include benzene, toluene, xylene, and other hydrocarbons like d-limonene and phenol.^65^ It is reported that the fractions of aromatic compounds increase in the TPO, while the aliphatic fraction decreases as the temperature increases.^18^

#### 4.1.2 TPO Fractional Content

As previously mentioned, the primary products of the pyrolysis of ELT include oil, which contains a range of chemical compounds such as aliphatic, aromatic, and other hydrocarbons,^66^ as indicated in Figure 12.^67^ To further understand these products, their relative content was studied by Wang et al.,^9^ which is graphed in Figure 13. It shows the relative content of the various chemical structures that are produced from the pyrolysis of ELT and its copyrolysis-pyrolysis with PS and PP.^64^ The data indicate that the pyrolysis of tires produces a high amount of aliphatic and aromatic hydrocarbons, where the addition of PS lowered the aromatic compounds to produce ketones, carbohydrates, and phenols.^65^ On the other hand, copyrolysis with PP produced a wide range of additional compounds like esters, aldehydes, and a small content of acids.^66^ These new products come from the decomposition of NR, BR, and SBR which is enhanced by adding plastic wastes such as PS and PP.

**Figure 12:**
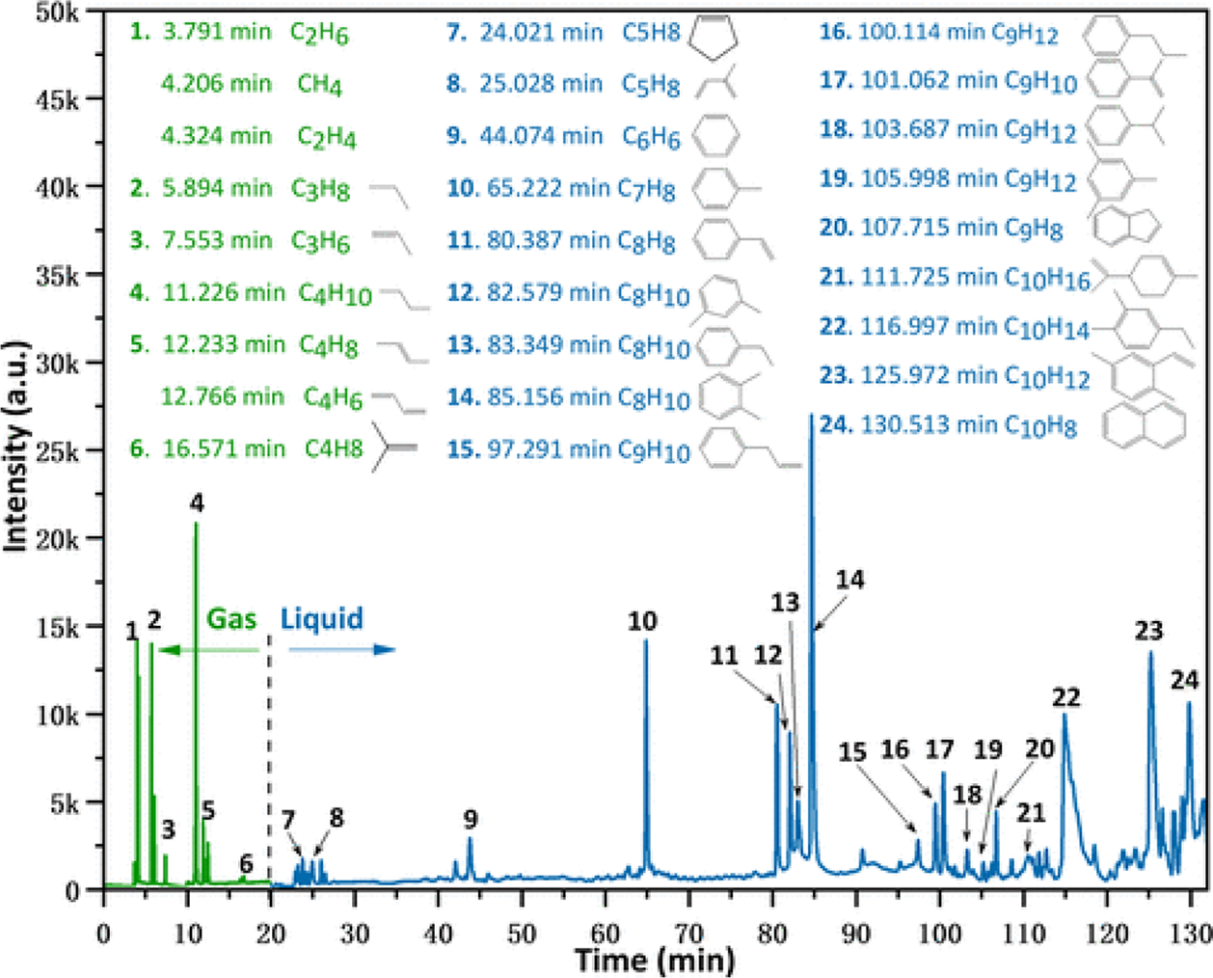
Chromatogram of ELT’s pyrolysis products at 775 K. Reproduced with permission from ref 67 available under the Creative Commons Attribution License (CC BY), Li et al., copyright [2022].

**Figure 13:**
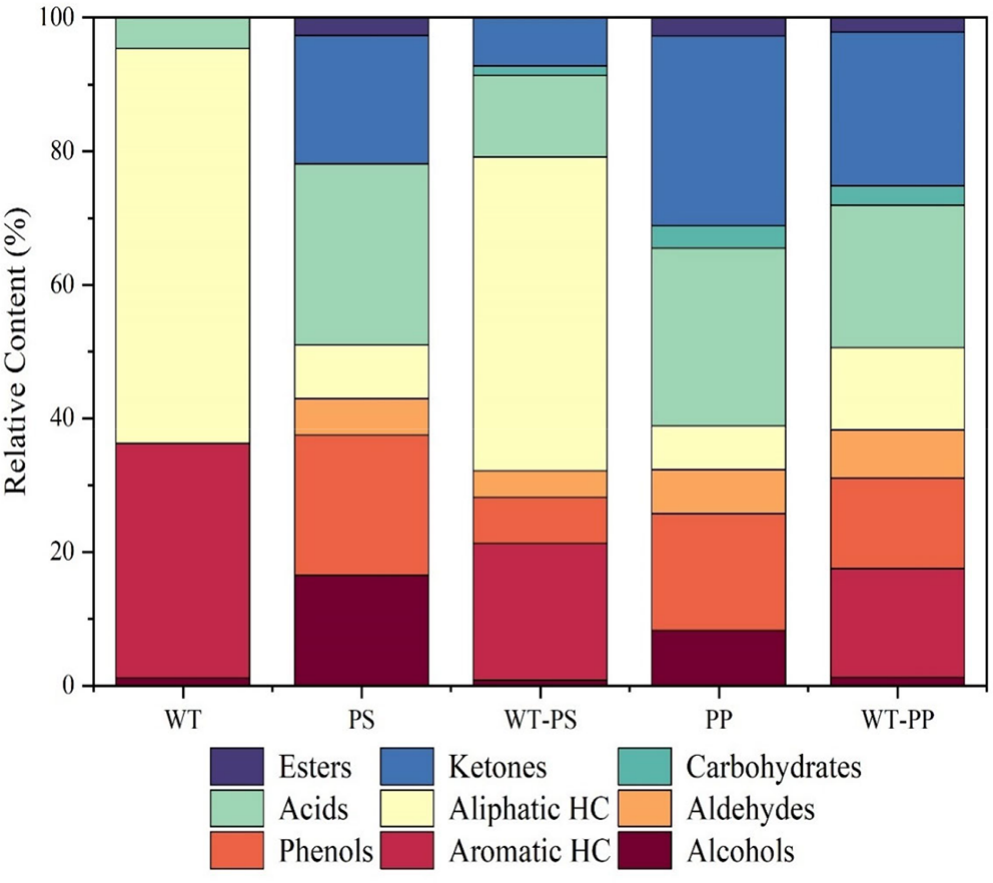
TPO’s relative fractional content for different hydrocarbons that emerge from the pyrolysis of WT,^9^ PS,^64^ and PP.^65,66^

#### 4.1.3 TPO Applications

Although the TPO exhibits a promising calorific value and hydrocarbon content, it cannot be used directly as a fuel in combustion engines or other applications due to its high sulfur content and acidity.^68^ Therefore, TPO is processed by hydro processing to improve its characteristics and properties. As a result, this oil can be converted using different chemical refinement processes to produce high-quality products, such as petroleum or diesel. Murugan et al.^69^ studied the combustion properties of a blended TPO-diesel mixture. It is reported that introducing this oil affected the performance of the engine. For instance, a marginal increase in the brake thermal efficiency was observed in the coblended mixture in reference to pure diesel. Moreover, the higher the blend ratio of oil to diesel, the greater its effect on the overall emission (NO*_*x*_*) of the engine.^70^ Furthermore, Yun et al.^71^ reported the hydrotreating process of TPO over highly dispersed Ni_2_P catalyst supported on SBA-15, where the results suggested a decrease in the carbon, sulfur, and nitrogen contents in the TPO after the hydrotreating process. This implies that TPO could be upgraded via catalytic hydrogenation reactions.

#### 4.1.4 Sulfur Removal

The primary challenge in utilizing this oil is its sulfur content, which can be reduced through hydro-desulfurization. This process entails removal of sulfur from the oil, which also eliminates any hazardous substances that contribute to the formation of S-bearing toxicants.^72^ Other techniques, such as oxidative desulfurization, serve to remove these harmful substances. The primary sulfur method is hydrogenation, which operates at elevated temperatures and pressures, where these conditions are used to increase the sulfur removal efficiency from the TPO mixture.^73^ This method operates using catalysts like Mo supported on aluminum oxides, where other metals can be doped into the catalysts, like nickel (Ni) or cobalt (Co), to improve the catalyst’s activity.^35^ The second approach is the oxidative method, which utilizes an oxidizing agent like hydrogen peroxide (H_2_O_2_) to react with sulfur and converts it to sulfides.^35^ Table 4 summarizes the operational parameters of these two desulfurization methods for the associated sulfur removal efficiency. The hydrogenation method generally incurs higher sulfur removal efficiency as illustrated by Jantaraksa et al.^74^

**Table 4:** Summary of the Operational Parameters of TPO Desulfurization Methods and Their Associated Sulfur Removal Efficiency

system-catalyst	conditions	sulfur removal	ref
oxidative-H_2_O_2_/Al2O3	80 °C for 240 min	81.0%	^75^
oxidative- H_2_O_2_/SZrO2	60 min using 1 wt % catalyst	60.0%	^61^
oxidative-Ca (OH)_2_/ H_2_O_2_	500 °C using 5 wt % catalyst	84.0%	^76^
hydrogenation-CoMo-SiO_2_–Al_2_O_3_	380 °C at 5 MPa	82.0%	^77^
hydrogenation-Pt/C, Ru/C	400 °C for 120 min using 15 wt % catalyst	90.0%	^74^

### 4.2 Char Product Analysis

#### 4.2.1 Char Composition

Figure 14 illustrates the char composition resulting from the pyrolysis of ELT, ranging from 20 to 50 wt % in three types of reactors: fixed bed, rotary kiln, and drop tube. Cunliffe and Williams^78^ analyzed the char content formed from pyrolysis of ELT in a fixed bed reactor. Along the same line of enquiry, Galvagno et al.^79^ analyzed ash formed in a rotary kiln reactor, where a higher volatile content was reported. Likewise, Conesa et al.^80^ used a drop tube reactor to report comparable ash and volatile contents in the char fraction. The main compounds within the char consist of high carbon content and low hydrogen and sulfur. The relative loads of C, H, and S contents thus depend on the type of ELT, the reaction conditions, and the utilized reactor.

**Figure 14:**
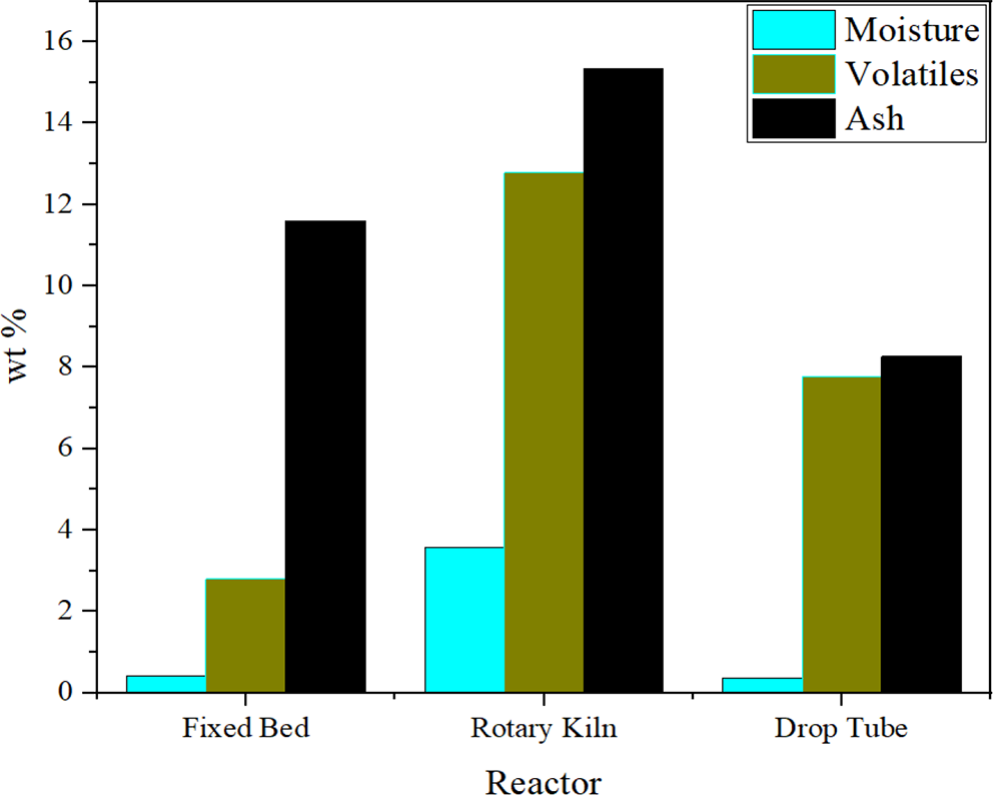
Char Compositions at 500 °C with different reactors: fixed bed data,^78^ rotary kiln,^79^ and drop tube.^80^

#### 4.2.2 Char Characterization

Previous sections illustrated that char production from the ELT pyrolysis ranges from 20 to 50 wt % of the overall conversion. Table 5 reports the properties and elemental compositions (proximate and ultimate analyses) of the produced char. The proximate analysis presents the weight percentages of moisture, volatiles, and ash in the produced char. The analysis revealed high ash and volatile contents, with a low moisture content ranging from 0.4 to 3.57 wt %. The volatile content was reported to be the highest by Li et al.^81^ when using a rotary kiln reactor, reaching a weight value of 16.14 wt %, while it was the lowest in the fixed bed reactor reported by Cunliffe and Williams.^78^ The produced char mostly consists of a high carbon content, as the ultimate analysis by Cunliffe and Williams presented.^78^ Regarding the hydrogen and nitrogen contents, their weight contribution is minimal compared to those of other materials. In the case of ELT’s char analysis, reporting the sulfur content proves very important from operational and environmental viewpoints. Conesa et al.^80^ reported the lowest sulfur content when using the drop tube reactor compared to the analysis studied by Li et al.^81^ using the rotary kiln reactor. Overall, the sulfur content is reported to have close values regardless of the reaction conditions since it comes from the manufacturing of tires, where this value ranges from 2 to 3 wt % of the overall tire. The calorific value of the char remains within ∼30 kJ/kg, reflecting the consistent chemical composition revealed by the elemental analysis.

**Table 5:** Properties of Char Produced from the Pyrolysis of ELT

reference	^78^	^80^	^81^	^79^
reactor	fixed bed	drop tube	rotary kiln	rotary kiln
temperature	500 °C	450 °C	500 °C	550 °C
proximate analysis (wt %)				
moisture	0.4	0.37	2.35	3.57
volatiles	2.8	7.78	16.14	12.78
ash	11.6	8.27	12.32	15.33
ultimate analysis (wt %)				
carbon	90.6	88.19	82.17	85.31
hydrogen	0.9	0.6	2.28	1.77
nitrogen	0.7	0.1	0.61	0.34
sulfur	2.3	1.9	2.32	2.13
chlorine	0.08			
calorific value (MJ/kg)	30.5	30.8	31.5	30.7

#### 4.2.3 Char Applications

The produced char from the pyrolysis of ELT showed a promising result as a substitute for carbon black, where several studies investigated the properties of the produced char and compared them to carbon black.^82^ As mentioned before, char accounts for more than 30% of the overall byproducts of the pyrolysis of ELT, and it originates from the continuous heating of hydrocarbons, which cause them to burn and decompose.^83^ Therefore, the produced char or the pyrolytic carbon black can be used for many industrial applications, most notably as an elastomer reinforcer, a metal coating, and a heavy metal adsorbent. Moreover, the resulting char as a source of carbon black in new tires reduces the environmental footprint of the tire manufacturing process.^84^ The problem with the char produced from the pyrolysis of tires containing higher ash and volatile compounds is related to natural carbon black. Furthermore, its structure and surface area are slightly different, which affects its application in certain industrial areas, or sometimes it needs to be processed to be compared to the natural carbon black.^85^

### 4.3 Gas Product Analysis

Figure 15 lists the gas compositions of the pyrolysis of ELT, where the composition is mainly dominated by hydrogen, carbon dioxide, carbon monoxide, methane, and hydrogen sulfide. These results are analyzed at a temperature of 600 °C with different reactors. Kaminsky et al.^30^ studied the reaction using a fluidized bed, where most of the composition in the gas was methane with a minor amount of hydrogen. Another study by Williams and Brindle^29^ used a fixed bed reactor while producing a low quantity of gaseous products that mainly contain methane, hydrogen, carbon dioxide, and a negligible amount of carbon monoxide. Additionally, Aylon et al.^86^ experimented the pyrolysis of ELT using a rotary kiln where H_2_S was reported. It is clear that H_2_S emerges from the reaction of the sulfur content with hydrogen atoms in a sequence of reactions.

**Figure 15:**
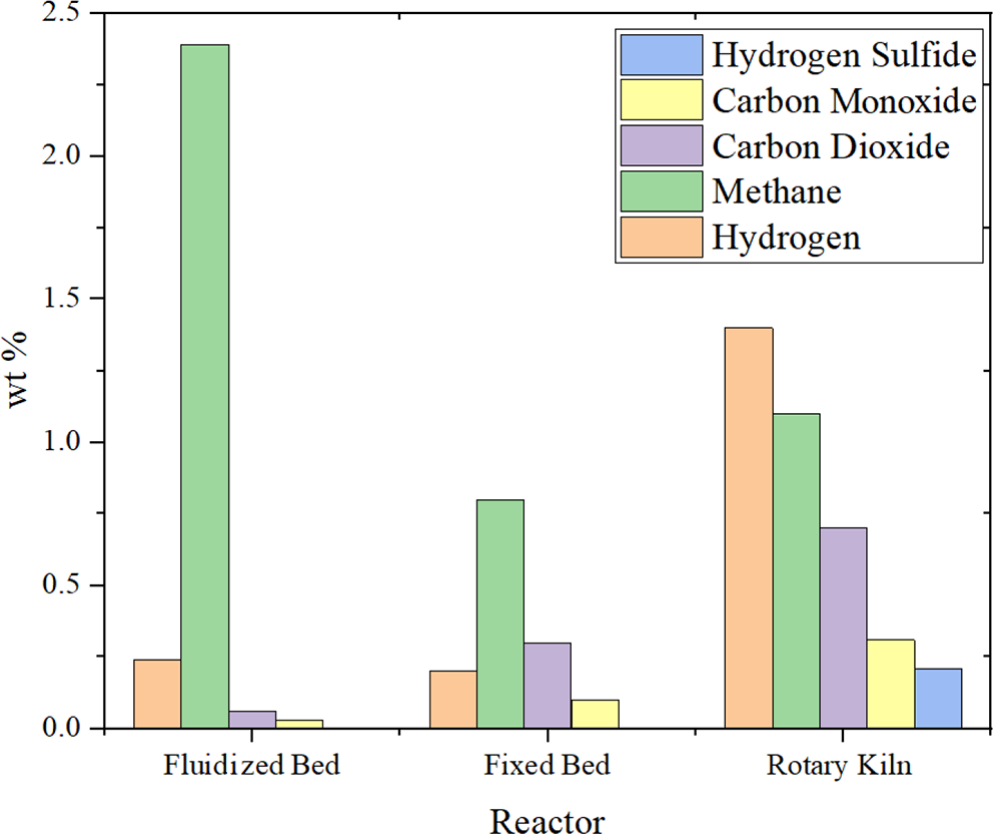
Gas compositions at 600 °C from the pyrolysis of ELT. Data were sourced from their respective references, fluidized bed,^30^ fixed bed,^78^ and rotary kiln.^79^

Table 6 summarizes the gas composition products from the tire pyrolysis reaction, with their corresponding fractions. Williams and Brindle^29^ and Kaminsky et al.^31^ investigated the pyrolysis reaction using a fluidized reactor at different temperatures. At the intermediate temperature range, Williams and Brindle.^29^ showed that the gaseous products mainly contain traces of carbon dioxide and carbon monoxide.^29^ At higher temperatures, Kaminsky et al.^31^ reported that hydrocarbon gases appear at a significantly higher temperature as compared to the William and Brindle experiment.^30^ In addition, the production of CO and H_2_S increases as the temperature increases. These gases are toxic and need further treatment since they impact the environment on a larger scale in reactors.^87^ The study of Conesa et al.^80^ reported higher loads of CO_2_ at higher temperatures. Furthermore, at even higher temperatures, methane production increased with no traces of H_2_S. Moreover, Galvagno et al.^79^ analyzed the ELT pyrolysis reaction in a rotary kiln reactor to report an increase of the production of hydrogen, methane, ethane, and higher hydrocarbons and H_2_S. A similar trend was also observed by Kaminsky et al.^30^ Last, Aylon et al.^86^ used a moving screw bed to mainly report H_2_S at a high temperature of 800 °C. Finally, Tables 7 and 8 present selected studies that investigated the pyrolysis of ELT and pyrolytic catalytic upgrading.

**Table 6:** Gas Composition from the Pyrolysis of ELT

		gas composition (wt %)								
reactor type	temperature (°C)	H_2_	CH_4_	C_2_H_4_	C_3_H_8_	C_3_H_6_	CO_2_	CO	H_2_S	references
fluidized bed	450	0.06	0.29	0.24	0.16	0.04	trace	trace		^18^
fluidized bed	600	0.59	2.9	1.6	0.76	2.2	1.8	1.6	0.38	^30^
fluidized bed	700	1.1	6.9	4.0	0.63	4.4	2.5	2.1	0.6	^31^
drop tube	450	0.05	0.65	0.59	0.09	0.22	3.37			^80^
drop tube	750	0.35	4.62	2.42	0.14	1.11	4.11	0.6		^80^
rotary kiln	600	1.4	1.11	0.67	0.67		1.81	1.32		^79^
batch	500	0.2	0.8	1.2	0.3	0.5	0.3	0.1		^29^
moving screw bed	600	0.7	3.6						1.4	^86^
moving screw bed	800	1.8	13.8						0.8	^86^

**Table 7:** Summary of Studies that Report Products, Used Reactors, and Operational Conditions on the Pyrolysis of ELT

		maximum product yield (wt.%)			
reactor type	reaction conditions (temperature, feed rate)	oil	char	gas	ref
fixed bed	300–700 °C; 5–80 °C/min heating rate; 50g tire	58.5%	26.4%	14.8%	^24^
fluidized bed	750 °C; 30 kg/h feed; tire pieces	31.9%	38.0%	28.5%	^30^
drop tube	450–1000 °C; 30 g/h feed	37.8%	35.2%	26.9%	^80^
rotary kiln	550–680 °C; 4.8 kg/h feed	38.12%	49.09%	2.39%	^79^
moving screw	600–800 °C; 3.5–8 kg/h feed	48.4%	39.9%	11.7%	^86^
rotary kiln	450–650 °C; 12–15 kg/h feed	45.1%	41.3%	13.6%	^81^
vacuum	500 °C	56.5%	33.4%	10.1%	^33^
vacuum	450–600 °C; 100g (batch)	47.1%	36.9%	16.0%	^34^

**Table 8:** Summary of Selected Studies that Deploy Catalysts to Enhance the Yields of TPO from the Pyrolysis of ELT

		maximum product yield (wt.%)			
catalyst	reaction conditions (temperature, feed rate)	oil	char	gas	ref
ZSM-5	430–600 °C; 10 °C/min heating rate; 1.4 mm tire	34.6%	38.0%	20.0%	^53^
Al_2_O_3_	500 °C; 10 °C/min heating rate; 18 mesh tires	33.1%	40.0%	21.2%	^36^
CaCO_3_	300–400 °C; 10 °C/min heating rate; 10 mm tire	32.23%	35.23%	32.52%	^59^
MgO	371–504 °C; Batch	35.1%	35.5%	8.6%	^58^
Ca (OH)_2_	500 °C; batch	40.0%	48.0%	12.0%	^26^
Ru/MCM-41	500–600 °C; micrometric machine	24.8%	44.5%	32.0%	^60^
Cu/HBETA	350–500 °C; 10 °C/min heating rate; 40 mesh tires	39.0%	40.5%	11.0%	^61^

## 5 ELT: Decomposition and Impacts

### 5.1 Other Techniques for the Decomposition of ELT

#### 5.1.1 Grinding

ELT grinding is the method of breaking the ELT structure into scraps with the help of high-technology mills at normal temperatures, which can be divided into multiple steps. First of all, the shredding of tires produces small rubber particles, but in this step, multiple grinding processes are completed to have a uniform structure of the rubber particles.^88^ However, this continuous grinding can generate heat from friction, resulting in rough surfaces because of oxidation and degradation. After that, the metals and steel materials are removed from these particles using magnets and vibrations, where the resulting steel can be utilized in the manufacture of nails, while the rubber can be used for producing new tires.^89^ Furthermore, additional grinding produces better rubber particles, where complicated techniques such as cryogenic and ambient grinding are used. These techniques use liquid nitrogen to change the rubber into a brittle material, where it can be fractured into finer particles.^90^ The only issue with this technique is that it uses liquid nitrogen, which increases the operational cost. Finally, the generated particles are screened to meet different standards. Magnetic separation uses a magnet to attract the metal components from the rubber, while the vibration method separates rubber and metal by their size, which will prevent larger metals.^91^ The other method is air separation, which utilizes an air stream to separate lighter components from the heavy ones, while water extraction is carried out by immersing the rubber in water to separate them from the metals. The steps for the grinding of ELT are summarized in Figure 16.^92^

**Figure 16:**
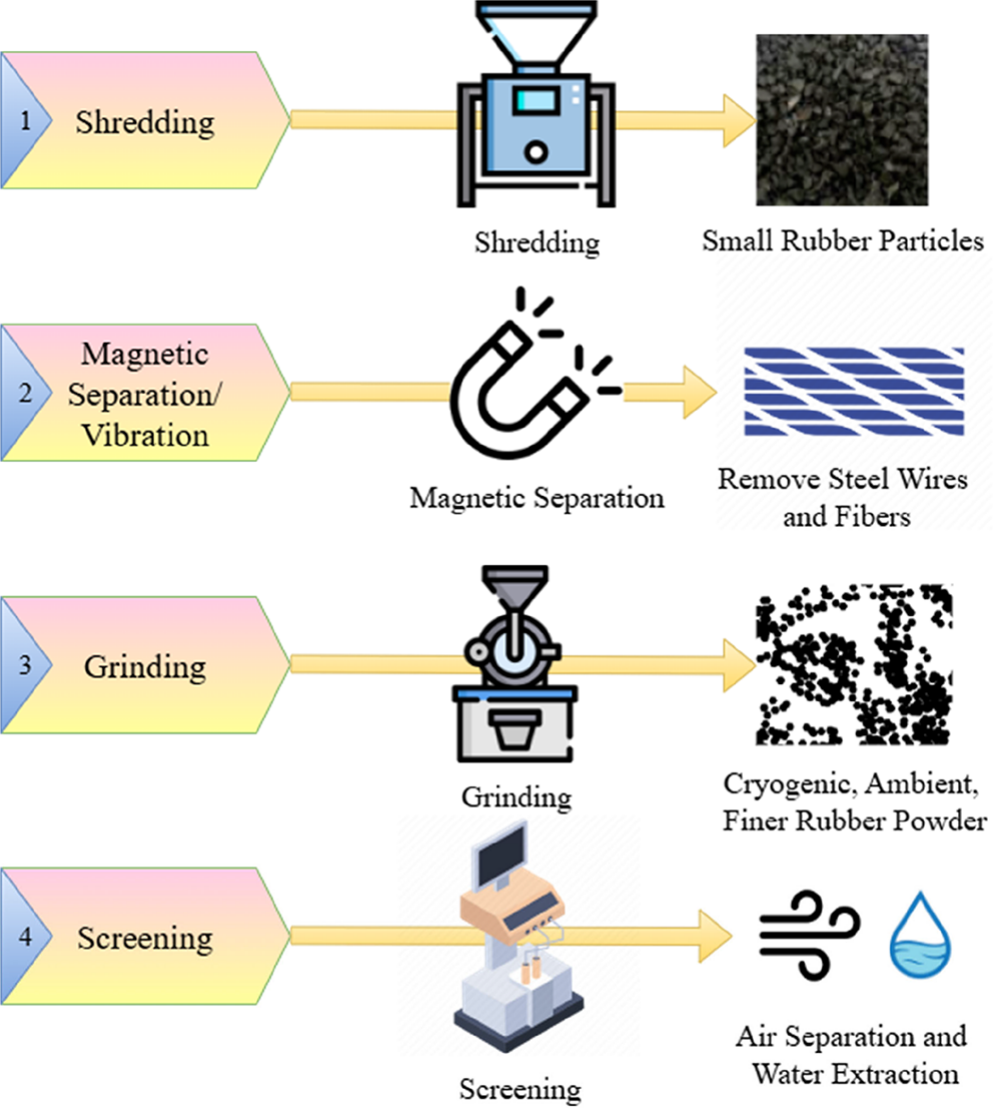
Four grinding steps of ELT.

#### 5.1.2 Gasification

Gasification is another method used for the decomposition of ELT comprising several distinct techniques, as stated by Fajimi et al.^93^ The first method is conventional gasification, which is a relatively new technology characterized by a simple operation and low cost. However, despite its common use, the method has notable disadvantages, including a lengthy processing time and potential environmental pollution. The second gasification method utilizes plasma to gasify the ELT sample at low temperatures.^94^ However, it is highly complex and requires significantly more energy when compared with other methods. As in the plasma-assisted method, hydrothermal gasification is an energy-consuming operation.^93^ Solar-assisted gasification deploys solar power to produce continuous heating.^95^ Cogasification employs multiple gasification methods to enhance the production of ELT products. However, this method is still in the initial stages of development, which needs further research and exploration.^92^

#### 5.1.3 Categories of Desulfurization Methods

Desulfurization methods can be grouped into physical, chemical, and biological methodologies, which entail various advantages and disadvantages^96^ The physical method does not use a chemical additive, and thus it is only effective in the removal of inorganic sulfur.^97^ It is often deployed as a pretreatment methodology for further deep and complete desulfurization. On the other hand, chemical desulfurization has the capacity to remove both organic and inorganic forms of sulfur.^96^ This method often leads to the production of high-quality products with low energy consumption.^98^ However, the chemical method involves the deployment of expensive chemicals and catalysts with aggressive chemical agents. Biological-based methods offer eco-friendly alternatives to physical and chemical desulfurization, leveraging the ability of microbes to metabolize sulfur compounds under controlled conditions.^99^ This is the eco-friendliest approach for desulfurizing the ELT as it is cheap and energy-efficient. The only downside of this technique is that it has slow reaction rates, and it needs nutrients to operate while producing products to be carefully managed.^92^

### 5.2 Environmental Impact of ELT Pyrolysis

#### 5.2.1 Environmental Impacts

The positive environmental impacts of ELT pyrolysis start with reducing waste in the landfill since it occupies a large area for storing, while it produces oil and gas that can be used as supplement to fuels.^100^ Furthermore, the pyrolysis operates in an oxygen environment that minimizes the formation of toxic pollutants like dioxins and GHG emissions. On the other hand, the pyrolysis of ELT produces considerable emissions of VOCs, PAHs, and sulfur oxides (SOx) that are generated from the thermal degradation of rubber additives and impurities as well as from incomplete combustion of gases.^1^ As explained throughout this review, the pyrolysis of ELT consumes considerable energy in operation since it operates at temperatures between 400 and 800 °C, and it produces solid char such as carbon black ash, and trace metals, which account for roughly 30–40% of the tire mass.^100^ Moreover, improper disposal of this residue poses risks to soil and water contamination through leaching of heavy metals. Therefore, strategies for using char in asphalt and construction materials are explored to mitigate these negative effects. Moreover, life cycle assessment has shown that if pyrolysis systems were well managed, they would have lower environmental impacts. These assessments consider input energy and product recovery. The positive and negative impacts of the ELT pyrolysis are summarized in Figure 17.

**Figure 17:**
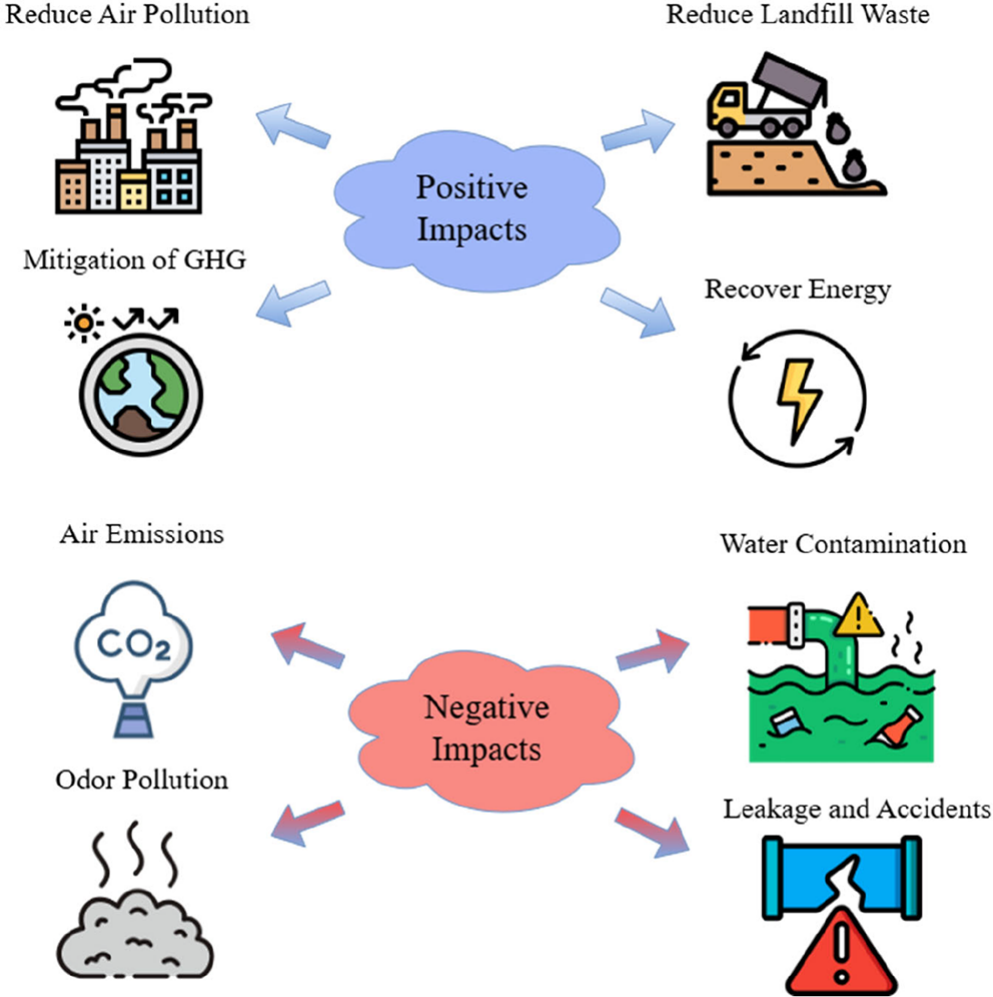
Environmental impacts of ELT pyrolysis.

#### 5.2.2 Mitigation Strategies

Mitigating the environmental impacts of tire pyrolysis requires comprehensive strategies focused on emission control, resource management, and waste handling. Advanced air pollution control systems, such as scrubbers and filters, can capture VOCs, particulate matter, and GHGs.^101^ Properly designed pyrolysis reactors with effective seals minimize leaks and improve the process efficiency. Byproducts like pyrolytic oil, gas, and char should be treated and refined to ensure safe reuse or disposal, with char detoxification methods addressing heavy metals and PAHs.^101^ Utilizing renewable energy sources for the high-temperature pyrolysis process reduces its carbon footprint, while strict adherence to environmental regulations ensures compliance and sustainability. Public awareness and worker training on safe practices can further minimize risks and enhance operational safety.^101^

#### 5.2.3 Emerged Pollutants from Uncontrolled Pyrolysis

Figure 18 specifies the different classes of pollutants that emerge from the uncontrolled pyrolysis reaction of ELT. Both VOCs and PAHs are among the most prolific toxicants that ensue from the pyrolysis of ELT. The aromatic structure of the rubber components in ELT facilitates formation of PAHs as described in our recent mechanistic review.^102^ SO_*x*_ and reduced sulfur species also form invariably from the pyrolysis of ELT.^102^ Toxic heavy metals, such as zinc and lead, from uncontrolled pyrolysis and open burning of ELT could contaminate water and soil, causing soil degradation. Finally, solid pollutants such as carbon black, char, ash, and fine particulate matter are also produced from the pyrolysis of ELT. A shown in Figure 18, pyrolysis of ELT releases a wide spectrum of pollutants with adverse ecological and health effects.

**Figure 18:**
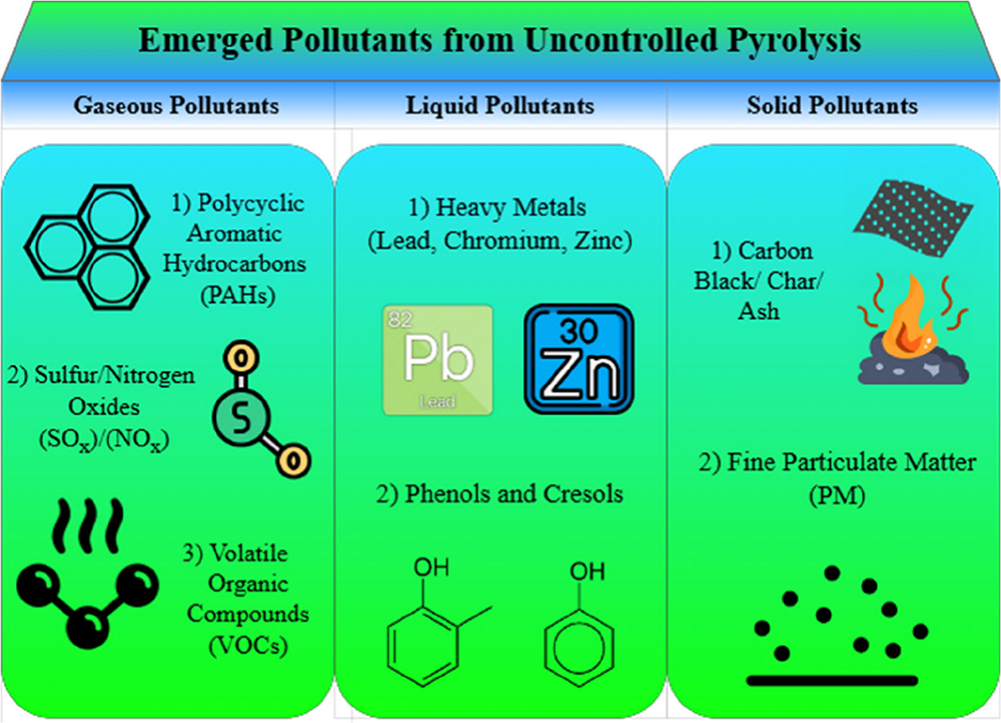
Emerging pollutants from uncontrolled pyrolysis.

### 5.3 Regulations and Future Trends

#### 5.3.1 Regulations and Policies

ELT pyrolysis is recognized as a sustainable method for handling ELT and potentially transforming it into valuable products. However, recycling and responsible disposal of ELT are governed by various regulations and policies worldwide to ensure compliance with sustainable waste management. For example, several states in the US collect additional fees at the time of vehicle registration to offset the anticipated cost of disposing of scrap tires.^2^ Collected fees are used to establish enforcement systems, to fund consumer education programs, and to provide grants or loans to tire recycling facilities. In the EU, environmental legislation prohibits the landfilling of whole or shredded tires.^103^ This has promoted efforts that aim to recycle and repurpose tires including pyrolytic operations. In the EU, it is the responsibility of the producer to collect 60% of its discarded tires, whereas 40% are to be collected and treated by private entities.^103^

#### 5.3.2 Future Trends

We envisage further improvement in the ELT pyrolysis toward the desired products may be realized through several potential future directions. Most notably, they are as follows:

1. Promoting the use of decentralized pyrolysis units that are deployed near waste sites. Such an approach reduces the transportation costs and emissions^104^
2. Surveying the promotional effects of various types of plastics on the quality of TPO via a systematic approach that investigates parameters such as the mixing ratio of plastics/ELT, the residence time, the temperature, and additives. This includes flow reactor experiments and online TGA-IR-GC experiments.
3. Implementing machine learning and artificial intelligence techniques to predict conditions that improve the performance of ELT pyrolysis and catalytic upgrading of TPO. For example, learnable AI tools could be used to underpin the optimum plastic/ELT ratios that maximize fuel-like compounds. The learning data set could be extracted from pertinent literature studies.
4. Performing detailed DFT computations to explore the very complex reaction pathways that operate during the decomposition of ELT’s individual components and their cointeractions.

## 6 Conclusions

The review aims to dissect the process of tire pyrolysis along with its use of converting waste into valuable products, including but not limited to pyrolytic oil, char, and gas. Moreover, it focuses on the significance it holds toward the economy and showcases different ways in which it can be implemented into different aspects, further allowing the economy to flourish. Still, there is the global issue of management of tire waste as it aggravates with time, and as the automobile industry continues to expand, so does the number of ELT. Being enthralled with tough competition in the automobile industry, organizations need to research effective methods to overcome this problem.

The paper emphasizes the significance of reactor design and its implications on the efficiency of pyrolysis and product distribution. It suggests why modular and vacuum systems are more efficient in oil output than other lower-end systems, such as fixed bed systems. Copyrolysis with plastic shows real-life scenarios where the tires can be contaminated with plastic, which can increase the number of new products formed and the amount produced. For instance, incorporating PS enhances the yield of oil produced and also changes the oil to be a high-quality oil with abundant aromatic hydrocarbons. This increases the need for both reactor and feedstock integration design systems to be customizable for specific requirements. As highlighted earlier, catalytic pyrolysis is critical in increasing the reaction rates, reducing the energy requirements, and improving the final product’s quality, indicating its significance. Using zeolite as catalyst also increased oil production, while calcium hydroxide aided in char formation during oil production pyrolysis. Additionally, strong dependency was noted regarding catalyst participation and the mechanism of the reaction/distribution of products, confirming the need for greater efficiency and greener catalytic materials. This perspective also considers the environmental impact that the pyrolysis of tires generates, which is beneficial because it offers the possibility of reducing the volume of deposits in landfills and presents cleaner fuels than those produced from direct combustion, which is traditionally employed. Some of the issues, however, that need to be addressed are the release of VOCs, the sulfur level in the pyrolytic oils, and pollution by toxic residues. Advanced air pollution control technology, renewable energy mixtures, and effective processing of byproducts are important steps that should be taken to maintain the sustainability of the process.

Finally, pyrolysis stands as an optimized treatment method to address tire waste, merging resource recovery and environmental preservation at the same time. What is lacking from these studies is the attention on reaction conditions; temperature, feed composition, and even type of reactor have a profound effect on results. Research should be targeting better energy use, creation of multifunctional catalysts, and further progress of the technology for industry. If those challenges are tackled, modifying the existing frameworks of the process will enable ELT’s pyrolysis to greatly enhance global waste management systems.

## Abbreviations

BXT: benzene-xylene-toluene
BR: butadiene rubber
ELT: end-of-life tires
GHGs: greenhouse gases
NR: natural rubber
PE: polyethylene
PP: polypropylene
PET: polyethylene terephthalate
PS: polystyrene
PAHs: polycyclic aromatic hydrocarbons
PVC: polyvinyl chloride
SBR: styrene butadiene rubber
SDGs: Sustainable Development Goals
TPO: tire pyrolysis oil
VOCs: volatile organic compounds
